# Measurement of temporal and spatial parameters of ice hockey skating using a wearable system

**DOI:** 10.1038/s41598-022-26777-9

**Published:** 2022-12-24

**Authors:** Aminreza Khandan, Ramin Fathian, Jason P. Carey, Hossein Rouhani

**Affiliations:** Department of Mechanical Engineering, University of Alberta, 10-368 Donadeo Innovation Centre for Engineering, 9211-116 Street NW, Edmonton, AB T6G 1H9 Canada

**Keywords:** Biomedical engineering, Mechanical engineering

## Abstract

Ice hockey is a dynamic and competitive sport that requires a high level of neuromuscular and cardiovascular function. An objective assessment of skating helps coaches monitor athletes' performance during training sessions and matches. This study aimed to estimate the temporal and spatial parameters of skating by proposing an optimized configuration of wearable inertial measurement units (IMUs) and validating the system compared to in-lab reference systems. Ten participants were recruited to skate on a 14 m synthetic ice surface built in a motion-capture lab. Eight original event detection methods and three more adopted from gait analysis studies were implemented to detect blades-off and skate-strikes. These temporal events were detected with high accuracy and precision using skate-mounted IMUs. Also, four novel stride length estimation methods were developed to correct the estimated skaters' position using IMUs' readouts. The stride time, contact time, stride length, and stride velocity were obtained with relative errors of 3 ± 3%, 4 ± 3%, 2 ± 6%, and 2 ± 8%, respectively. This study showed that the wearable IMUs placed on skates and pelvis enables the estimation of temporal and spatial parameters of skating with high accuracy and precision, which could help coaches monitor skaters' performance in training.

## Introduction

Ice hockey requires high levels of aerobic and anaerobic fitness, well-coordinated body motion, and efficient functioning of the neuromuscular and cardiovascular systems^1–4^. Players with higher neuromuscular and cardiovascular abilities, capable of starting quickly and skating at higher speeds, are more likely to possess the puck and win face-to-face competitions in matches^5^. Accurate assessment of hockey players' skating movements during training sessions can help coaches continuously monitor players' performance with the aim of improving it during training. Spatial [e.g., stride length (SL) and velocity (SV)] and temporal [e.g., stride time (ST) and ice contact time (CT)] parameters of skating serve as mobility biomarkers^6,7^ and are recognized as significant metrics to characterize any repetitive activity like forward ice striding. These parameters, traditionally, were obtained in human motion laboratories using stationary motion-capture (MoCap) systems. However, the application of these instruments is limited since they are not available in every ice rink, and their captured volume is confined to a small part of the ice rink, which can disrupt the natural skating patterns of ice skaters^8–10^. Thus, wearable and garment-embedded technologies are preferable for on-ice skating performance assessments^11–13^.

Buckeridge et al. used a portable system composed of accelerometers, EMG modules, and force sensors to assess on-ice hockey player performance^14^. Also, Stetter et al. studied the feasibility of using wearable accelerometers to identify skating parameters such as ST and CT to differentiate players in terms of their skill level^5,15^. However, these studies investigated the skating parameters using 3D accelerometers rather than inertial measurement units (IMU). IMU has been applied to measure human motion for clinical outcome evaluation^16–18^, sport biomechanics evaluations^19–25^, and movement modalities detection^10,26^. They have the potential to obtain temporal and spatial parameters during hockey skating.

The computation of the temporal and spatial parameters, in the first step, requires the detection of skating temporal events. The accuracy of event detection using IMUs can vary significantly depending on the extraction method used^27,28^. The second step is to estimate the participant's trajectory in each stride necessary to calculate the spatial parameters^29^. Finally, temporal and spatial parameters can be calculated by the detected temporal events and the participants' trajectories. Participants' trajectory can be calculated using double-time integration of the participant's acceleration in a global reference frame. However, due to the cumulative error in the numerical integration of IMU readings, the obtained trajectory can be drifted and erroneous^30^. There are two types of error in calculating the stride length: the noise on the acceleration time series and the drift in the sensor orientation (used for double-integration of acceleration to estimate trajectory) obtained by the gyroscope readouts. In gait analysis application, it has been suggested to correct foot velocity and, subsequently, foot trajectory time series by assuming zero velocity and minimum foot height during foot-flat periods^6^. However, a similar period to the foot-flat with zero velocity in all directions is absent in ice hockey skating strides, making foot velocity and trajectory estimation more challenging in hockey skating than on-land gait. This study addresses these challenges toward improving the accuracy of on-ice measurement of spatial and temporal parameters of ice skating using a set of IMUs fixed on the participant's skates, shanks, and pelvis on a synthetic ice surface. Synthetic ice surface as an alternative to real ice with comparable forward skating mechanism^31^ has the potential to be used in in-lab testing and training, particularly where ice access is limited.

The objective of this study is to: (1) detect temporal events of skating using skate-mounted IMUs, (2) estimate the skate trajectory using IMUs, (3) calculate the temporal and spatial parameters of skating using the obtained temporal events and corrected skate trajectory, and (4) experimentally validate the obtained results against those measured by in-lab motion-capture systems on a synthetic ice surface.

## Methods

### Participants

Ten able-bodied individuals (age 25 ± 8 years, height 179 ± 9 cm, body mass 78 ± 11 kg; mean ± standard deviation (SD) among participants, six male and four female) were recruited to participate in this study. All participants were free from injury and capable of skating comfortably. The study was approved by the research ethics board of the authors' current institution (Pro00092821), and all methods were performed in accordance with the relevant guidelines and regulations. All participants were informed of the experimental procedures and gave informed written consent before the test.

### Experiments

**Figure 1:**
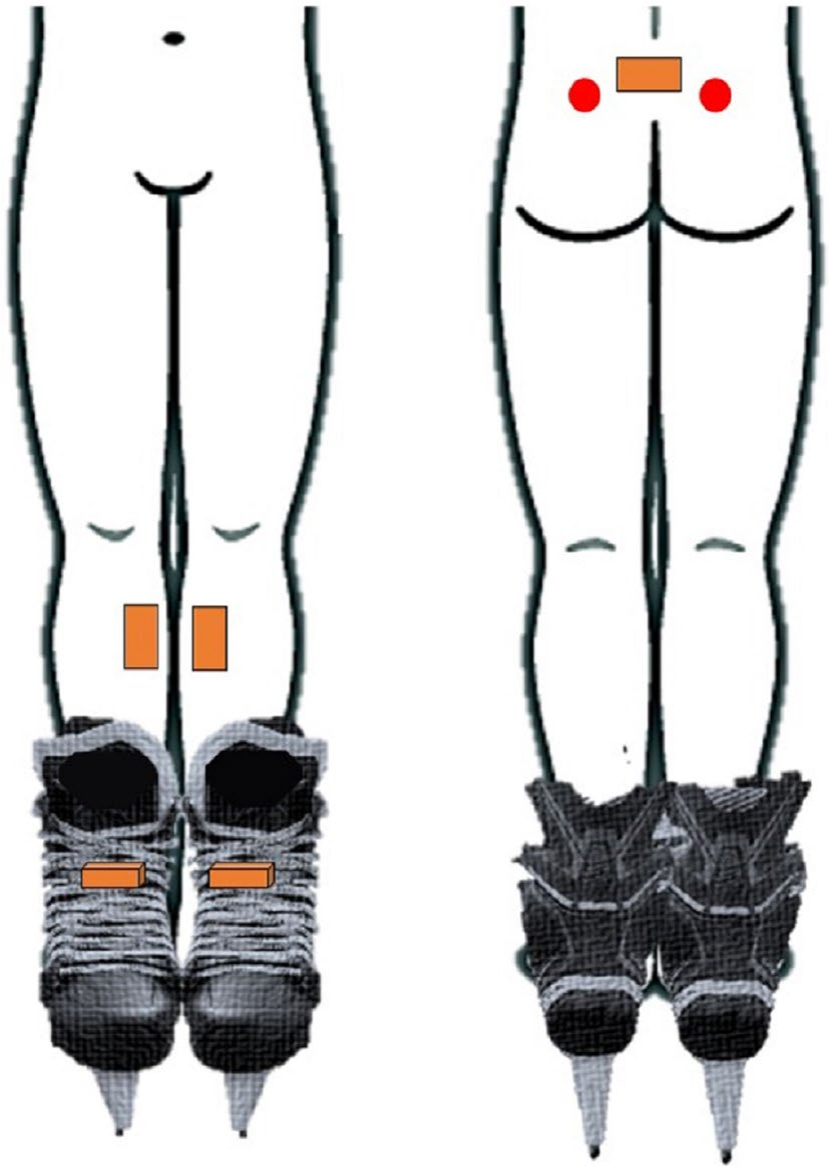
Five IMUs (orange boxes) were placed on the participants' pelvis, shanks and skates. Also, two pressure insoles placed in the skates and two retro-reflective markers on the PSISs (red circles) were used as a reference system for temporal event detection and stride length estimation, respectively.

Tests were carried out at an indoor synthetic ice rink ($14\times 2 {\mathrm{m}}^{2}$). Five IMUs (Xsens Technologies^32^, NL, full-scale ranges are: acceleration: $\pm 160 \mathrm{m}/{\mathrm{s}}^{2}$, angular velocity: $\pm 2000 \mathrm{deg}/\mathrm{s}$, and magnetic field: $\pm 1.9 \mathrm{Gauss}$) were placed on the pelvis, shanks, and two skates of the participants. No sensor-to-segment calibration was used, and sensors' readouts were directly used to extract the temporal events. The participants were asked to wear tight-fitting pants or shorts, and the sensors were placed on the skates and skin of the participants or on the fitted pants to minimize the garment-to-skin motion artifact. Two reflective markers were placed on the two posterior superior iliac spines (PSIS) of the body, as demonstrated in Fig. 1. As a reference system for temporal event detection, plantar pressure insoles (Pedar-X^33^, Novel, DE) were placed in the skates (Fig. 1) to measure the ground reaction force magnitudes and thus detect the instances of skate contacts on the ice. The pressure insoles were calibrated at the beginning of each session as a standard practice instructed by the manufacturer to remove the offset error. As the reference system for spatial parameters, 12 motion-capture cameras (eight Vero and four Bonita, Vicon^34^, UK) were used to track the trajectory of retro-reflective markers. After 10 s of standing still, the participants skated forward alongside the synthetic ice rink for 14 m. At the end of the forward skating trial, they also stood for 10 s quietly. During each trial, the IMUs, motion-capture cameras, and pressure insoles recorded their motions and ground reaction forces simultaneously. All the systems' sampling frequencies were 100 Hz, and each skating trial was repeated five times.

### Temporal event detection

**Figure 2:**
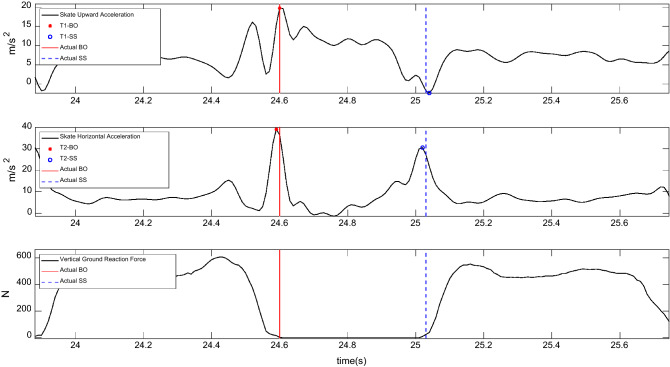
Exemplar recorded time series of skate upward and horizontal acceleration measured by the skate-mounted IMU in a skating trial. The figure also shows the temporal events (SS: skate strike and BO: blade off) detected by algorithms T1 and T2 and the actual events detected using the pressure insoles (gold-standard), based on a 5-N threshold on the vertical ground reaction force. Actual SS and BO were obtained from the first data points after the vertical ground reaction force passed the 5-N thresholds.

First, the IMU readouts measurements were filtered using a low pass 4th order Butterworth filter using 15 Hz. We developed eight original methods to detect skate strike (SS) and blades-off (BO) instants using the IMUs' readouts. In addition to the originally proposed methods of temporal event detection, described in Table 1, three highly-cited gait event detection methods in literature^28,35,36^ were adopted and implemented to detect the temporal events in skating. These 11 methods were implemented in MATLAB (Mathworks, USA) and obtained the events during five trials of each participant. These detected SS and BO were validated against those detected using the pressure insoles, with a 5 N threshold (Fig. 2).

**Table 1:** Description of the methods originally proposed (T1–T8) and adopted from the literature and modified (T9–T11) for detection of Skate Strike (SS) and Blade off (BO) instants during forward skating using the readouts of IMUs placed on the shanks or skates. Similar to the original gait analysis studies (T9–T11), it was assumed that the participant starts skating from a stationary position, and thus, starts by a BO (toe-off in original studies). Therefore, the odd negative peaks indicate BO (toe-offs in original studies), and the even peaks indicate SS (heel-strike in original studies).

Methods	Time series used	Event	Features used in time series
T1	Upward acceleration of skate	BO	Positive peaks of the time series
		SS	The minimum of the time series occurred between two consecutive BOs
T2	Horizontal acceleration of skate	BO	The minimum of the time series occurred before the positive peaks
		SS	The maximum of the time series occurred between two consecutive BOs
T3	Upward velocity of skate	BO	Negative peaks of the time series
		SS	Positive peaks of the time series
T4	Norm of skate acceleration	BO	The last minimum of the time series occurred before the dominant positive peaks
		SS	The maximum of the time series occurred between two consecutive BOs
T5	Shank angular velocity	BO	Negative peaks in the sagittal plane angular velocity
		SS	Positive peaks in the frontal plane angular velocity
T6	Norm of shank angular velocity	BO	The minimum of the time series occurred before the dominant peak
		SS	The minimum of the time series occurred between two consecutive BOs
T7	Norm of skate acceleration	BO	Odd numbered positive peaks in wavelet coefficients in the highest energy concentration frequency obtained by a CWT (continuous wavelet transform) utilizing Coiflet wave shape
		SS	Even numbered positive peaks in wavelet coefficients in the highest energy concentration frequency obtained by a CWT utilizing Coiflet wave shape
T8	Norm of skate acceleration	BO	Positive peaks in the 3rd approximation of the time series in a DWT (discrete wavelet transform) using Coiflet wave shape
		SS	The minimum of the time series occurred between two consecutive BOs
T9^21^	Shank angular velocity	BO	Odd-numbered negative peaks of the time series
		SS	Even-numbered negative peaks of the time series
T10^22^	Norm of skate acceleration	BO	Odd-numbered negative peaks of the time series
		SS	Even-numbered negative peaks of the time series
T11^19^	Norm of skate acceleration	BO	Local positive peaks of the time series
		SS	Local negative peaks of the time series

### Skater's stride length estimation

The spatial parameters were calculated using the IMUs placed on the skates, shanks and pelvis. Any inherent signal bias in the sensor readouts is prone to be accumulated in the integration process and corrupt the obtained velocity and trajectory of the skater. In the gait analysis application, the zero-velocity update (ZUPT) technique has been used to force the foot velocity to be zero during the stationary foot-flat period in each stride^6^. However, during contact time in ice skating, the skate keeps sliding on ice, making the ZUPT algorithm impractical in this application. In this study, four novel methods were proposed to eliminate the accumulating errors in the calculated velocity and trajectory of skaters:

(i) The accelerometer readout was transferred into the north-east-up (NEU) reference frame^37,38^, using the IMU orientation obtained by the Xsens software package,
(ii) The gravitational acceleration was subtracted from the transformed acceleration to obtain the free acceleration of the sensor in the NEU frame,
(iii) The skater's velocity was obtained using time integration of the free acceleration. The obtained velocity, however, was not zero in the resting periods due to the accumulated error of the IMU readouts,
(iv) The accumulated error was removed, and the obtained velocity and acceleration time series were corrected using the assumption of zero velocity and acceleration in the resting periods. To this end, four original methods were developed and implemented. These methods removed sensors' acceleration bias (S1), acceleration bias and estimated noise profile (S2 and S3), and estimated velocity error time series (S4) using the assumption of zero acceleration and velocity in the resting periods (Table 2),
(v) The corrected velocity was used to obtain the skater position trajectory used to obtain the SL for each stride (see “Temporal and spatial parameters estimation” below).

All these four algorithms (S1–S4) used IMU readouts during the resting period. The effect of resting period duration was investigated on the stride length estimation error to recommend a minimum resting period duration. To this end, IMU readouts collected during time windows from 0.1 to 10 s, by the increment size of 0.1 s, out of the two 10-s resting periods originally considered for data collection before and after the motion, were used in S1–S4. Then, the shortest resting period duration that would not significantly affect the stride length estimation error compared to longer resting periods was explored.

**Table 2:** The skater's velocity and trajectory are obtained by time integration and double-time integration of the free acceleration obtained by the IMUs. Methods were originally proposed (S1–S4) to remove the drift in these integration processes.

Method	Procedure
S1	(i) Calculate the mean of free acceleration value in the 0.5-s window resting period at the beginning of the trial right before the motion and subtract its representation in sensor frames from the raw acceleration time series and store it as corrected acceleration time series (ii) Calculate the corrected free acceleration (during motion) using the corrected acceleration time series (output of S1.i) and the sensor orientation (iii) Obtain trajectory using double-time integration of the corrected free acceleration
S2	(i) Similar to S1.i (ii) Generate white Gaussian noise using a MATLAB *wgn* function (input arguments: the signal-to-noise ratio: − 15 decibels (dB), and its length equal to the sensor acceleration time series). Then scale it to match the corrected sensor acceleration amplitude range in the selected resting period. Afterward, subtract the obtained output from the corrected sensor acceleration time series and store it as corrected acceleration time series (iii) Calculate the corrected free acceleration (during motion) using the corrected acceleration time series (output of S2.ii) and the sensor orientation (iv) Similar to S1.iii
S3	(i) Similar to S1.i (ii) Apply DWT (discrete wavelet transform) to the corrected sensor acceleration (output of S3.i). Then, remove the first three DWT's details coefficients utilizing Coiflet wavelet basis function from the whole time series and store it as corrected acceleration time series (iii) Calculate the corrected free acceleration (during motion) using the corrected acceleration time series (output of S3.ii) and the sensor orientation (iv) Similar to S1.iii
S4	(i) Calculate velocity using time integration of free acceleration (ii) Estimate the noise profile using two resting periods at the beginning and end of each trial (right before and after the motion and with a length of at least 3 s), using piecewise cubic Hermite interpolating polynomial curve fitting and subtract it from the output of S4.i and store it as corrected velocity (iii) Obtain trajectory using time integration of corrected velocity (output of S4.ii)

### Temporal and spatial parameters estimation

ST was computed from one SS to the subsequent SS of the same skate, and CT, the time when the skate is in contact with the ice, was calculated from one SS to its subsequent BO. Also, SL in each stride was calculated using the estimated sensor trajectory. SL, then, was defined as the two norms of a 2D vector containing the travel distance in the mediolateral ($${p}_{ML}$$) and anteroposterior ($${p}_{AP})$$ directions in each stride (Eq. 1).

1 $$SL= \sqrt{{p}_{ML}^{2}+{p}_{AP}^{2}.}$$

SL was calculated using each of the four proposed velocity and acceleration correction methods. Finally, SV is calculated based on the corrected velocity time series obtained by the stride length estimation methods.

### Data analysis

**Figure 3:**
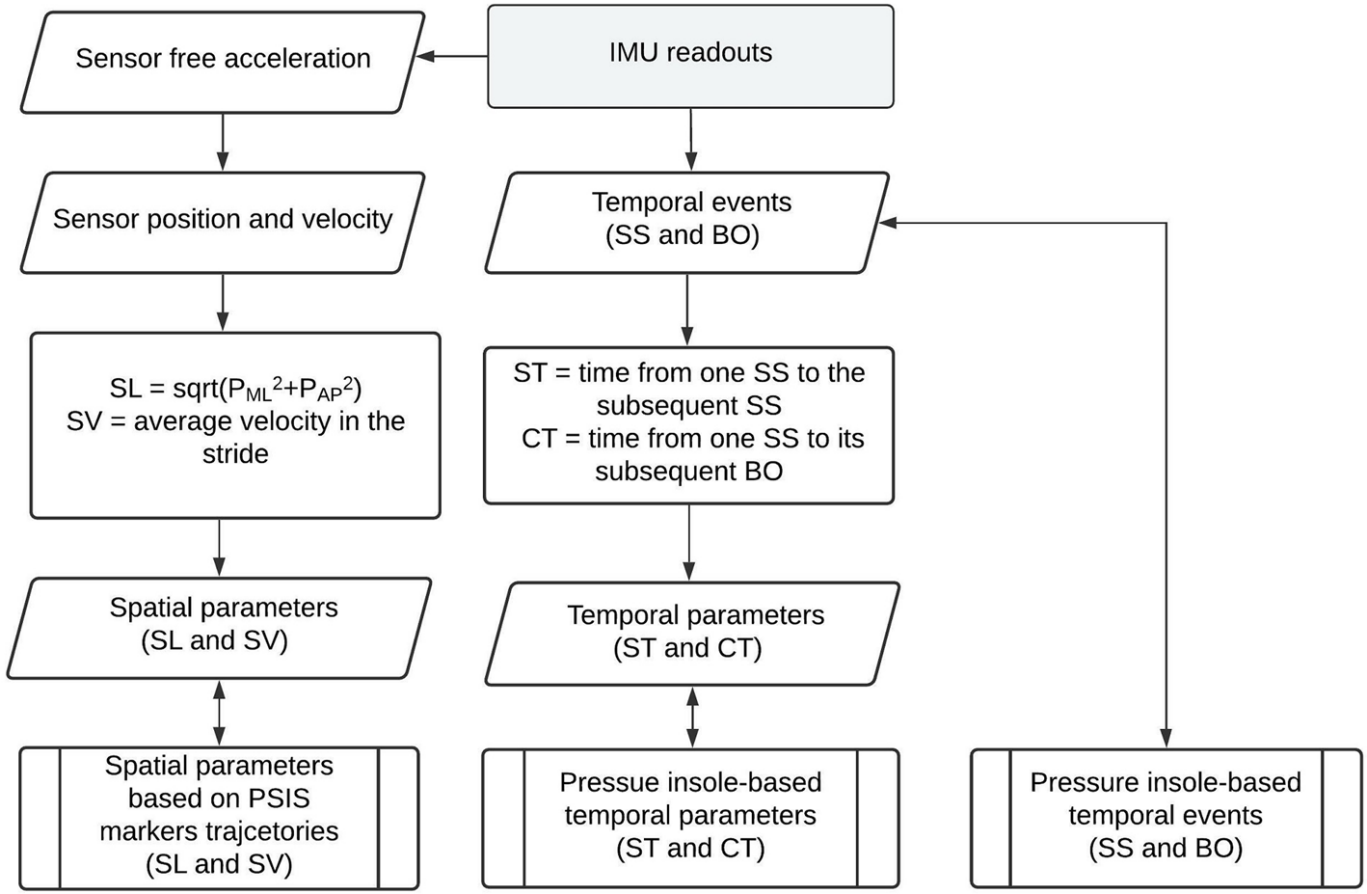
Flowchart of the measurement of temporal and spatial parameters of ice skating using IMU readouts and comparing it with the ones obtained by stationary in-lab reference systems. Here, sqrt stands for square root, and ${p}_{ML}$ and ${p}_{AP}$ respectively are the travel distance in the mediolateral and anteroposterior directions in each stride.

To compare the ability of the proposed methods against pressure insole in detecting the temporal events, the accuracy (mean) and precision (SD) of the errors were computed. To compare the SL estimated by the methods described in “Skater's stride length estimation”, the mean and SD of the relative error in addition to the error against the camera recording were calculated. There was no significant difference between the stride length obtained based on the markers on the pelvis and that obtained based on markers on the skate (rank-sum test (r = 0.98) indicated a failure to reject the null hypothesis at the 5% significance level). Additionally, unlike the markers on skates, markers on the pelvis have smoothers motion and would be a reliable reference for stride length measurement during each stride. Therefore, the reference SL is taken as the average travel distances of the PSIS markers in a stride. Finally, the set of best temporal event detection and SL estimation methods were selected to estimate the temporal and spatial parameters using IMU readout. These parameters were cross-validated against those calculated by in-lab reference systems recordings presented as the mean and SD of the relative error. Furthermore, a Bland–Altman plot modified for repeated measures^39,40^ has been provided to explore the agreement between the temporal and spatial parameters obtained by IMUs and those obtained by the reference parameters using Stata Statistical Software: Release 17^41^. Figure 3 shows the flowchart to calculate the temporal and spatial parameters using IMU readouts and validate the obtained parameters against the ones obtained by pressure insole data and camera recordings.

### Approval for human experiments

This study was approved by the research ethics board of the authors' current institution (Pro00092821), and all methods were performed in accordance with the relevant guidelines and regulations. All participants were informed of the experimental procedures and gave informed written consent before the test.

## Results

### Temporal event detection

In total, 184 SS and 186 BO were identified and then compared with those obtained by the pressure insole. The finding from method T4 revealed that temporal event detection with an average 0.01-s error using IMU readouts is achievable, equivalent to one sampling period (Table 3). Also, T9, adopted from gait analysis studies, obtained comparably 'accurate' results in skating event detection applications, i.e., an average error of 0.04 s and 0.05 s for detecting SS and BO, respectively. Yet, other event detection methods adopted from gait analysis studies (T1–T8, T10, T11) could not detect temporal events, particularly SS, with high precision, as indicated by the relatively high SD of the obtained errors. The mean $\pm$ standard deviation (SD) of the errors among study participants, reported in Table 3, suggest that the most effective methods in finding SS events were T1 ($0.00\pm 0.03 \mathrm{s}$), T2 ($-0.01\pm 0.03 \mathrm{s}$), and T4 ($-0.01\pm 0.04 \mathrm{s}$). Also, T3 ($-0.00\pm 0.05 \mathrm{s}$), T2 ($-0.05\pm 0.04 \mathrm{s}$), and T1 ($-0.03\pm 0.08 \mathrm{s}$) were more effective in detecting BO events in skating (Table 3). The negative errors indicate the SS or BO were detected before the actual event identified by the reference system (i.e., pressure insoles).

**Table 3:** Accuracy and precision of the developed and adopted methods in detecting 184 Skate strikes (SS) and 185 Blades offs (BO). The results are expressed as mean and standard deviations (SD) of the errors (in second, across participants) obtained by all methods described in Table 1 for detected temporal events using IMUs against those obtained using pressure insoles.

Methods	SS	BO
T1	$0.00\pm 0.03$	$-0.03\pm 0.08$
T2	$-0.01\pm 0.03$	$-0.05\pm 0.04$
T3	$-0.17\pm 0.09$	$0.00\pm 0.05$
T4	$-0.01\pm 0.04$	$-0.10\pm 0.04$
T5	$0.03\pm 0.20$	$0.09\pm 0.03$
T6	$-0.12\pm 0.06$	$-0.05\pm 0.07$
T7	$-0.19\pm 0.11$	$-0.06\pm 0.05$
T8	$-0.03\pm 0.04$	$-0.08\pm 0.04$
T9	$0.04\pm 0.18$	$0.05\pm 0.22$
T10	$0.04\pm 0.29$	$-0.09\pm 0.13$
T11	$-0.25\pm 0.23$	$-0.05\pm 0.04$

### Stride length estimation

The skaters' speed was $1.71\pm 0.61 \mathrm{m}/\mathrm{s}$ and ranged from 0.88 to 2.63 m/s among this study's participants. The relative errors of SL estimation using IMU readouts based on the methods described in Table 2 compared to the ones obtained by motion-capture cameras were investigated. S1–S3 required only a 0.5-s resting period right before the motion to correct the velocity, and longer resting periods did not enhance their performance (rank-sum test (r = 0.96)). They decreased the SD of the relative error to the range of 19–25% (compared to 47% when no correction method was implemented) when the free acceleration of pelvis-mounted IMU was used (Table 4). On the other hand, the velocity correction method (S4) required at least two 3-s resting periods right before and after the motion to correct the velocity, and longer resting periods did not enhance its performance [rank-sum test (r = 0.99)]. However, S4 was able to decrease the relative error from $7\pm 47\%$, obtained without any correction, to a range of $2\pm 6\%$ based on pelvis IMU readout (Table 4). In this method, the velocity was corrected by making the velocity time series to be zero in the resting periods.

**Table 4:** The mean and standard deviation (mean ± SD) for errors and relative errors of the stride length (SL) obtained by IMUs' readout against the SL calculated based on markers trajectory captured by the motion-capture cameras. The errors were calculated with and without applying the velocity correction methods (S1–S4, described in Table 2) using IMUs placed on the participants' pelvis, shank, and skates.

Method	Error (cm)			Relative error (%)		
	Pelvis IMU	Shank IMU	Skate IMU	Pelvis IMU	Shank IMU	Skate IMU
No correction	$-39\pm 109$	$-6\pm 58$	$-1\pm 106$	$-7\pm 47$	$-4\pm 26$	$-2\pm 41$
S1	$14\pm 53$	$14\pm 51$	$23\pm 47$	$9\pm 19$	$6\pm 22$	$9\pm 17$
S2	$14\pm 53$	$12\pm 49$	$19\pm 42$	$6\pm 23$	$6\pm 21$	$8\pm 16$
S3	$14\pm 53$	$14\pm 52$	$24\pm 47$	$7\pm 25$	$6\pm 23$	$9\pm 17$
S4	$3\pm 14$	$15\pm 30$	$-12\pm 40$	$2\pm 6$	$7\pm 13$	$-6\pm 14$

### Temporal and spatial parameters

**Figure 4:**
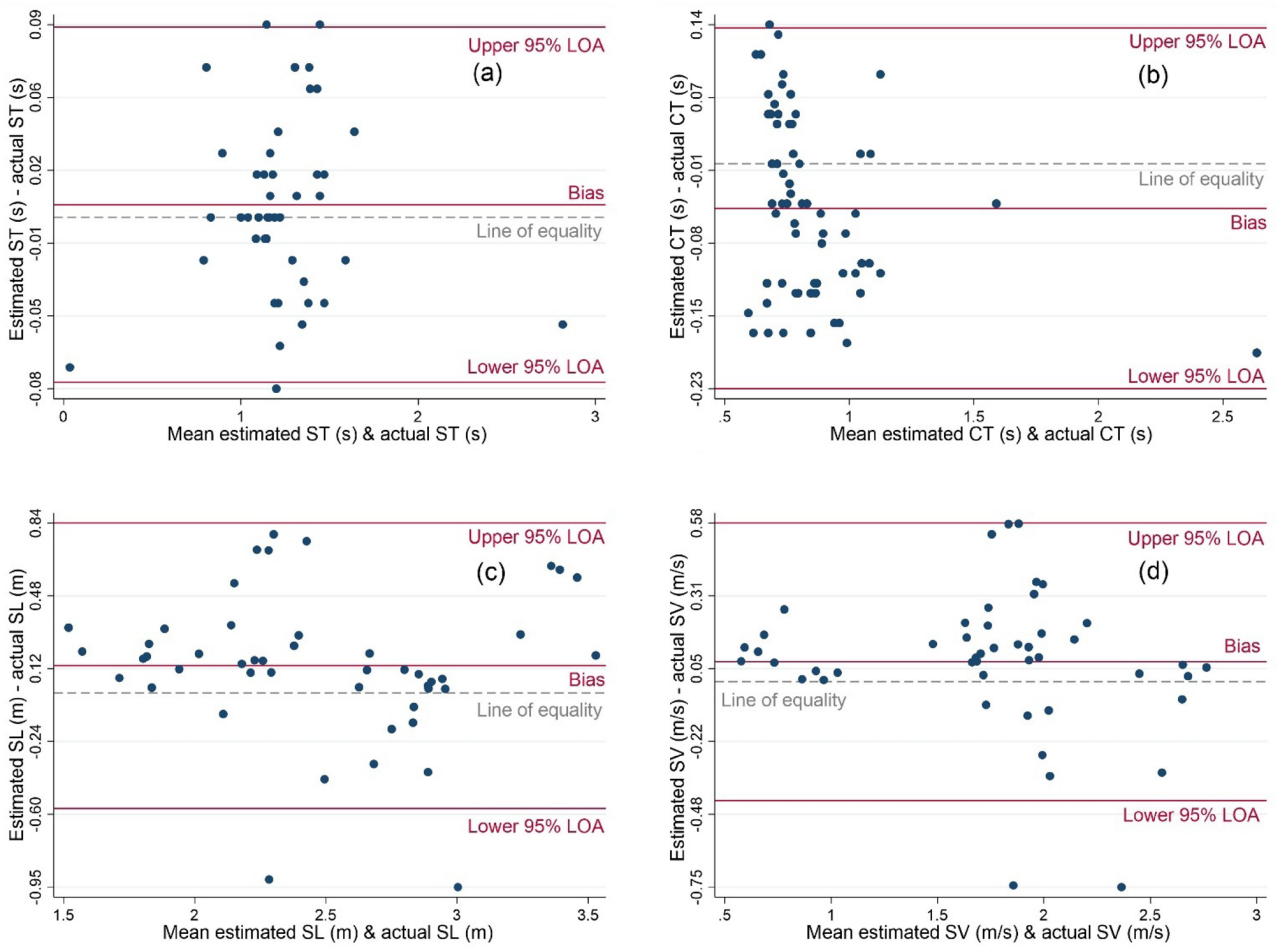
Bland–Altman plot modified for the repeated measures plotted for the ST (**a**), CT (**b**), SL (**c**), and SV (**d**) "estimated" by the IMU readouts against the "actual" ones obtained by the reference systems (pressure insoles for ST and CT, and motion capture system for SL and SV). Also, the lines of bias, upper 95% LOA (limits of agreement), lower 95% LOA, and the line of equality (i.e., line of zero error) are shown. The plot suggests no apparent relationship between the errors of the IMU-based developed methods and the magnitude of the temporal and spatial parameters.

ST and CT were calculated based on the temporal events identified by T1 and T3: the best methods in detecting SS and BO, respectively. Also, SL was calculated based on the trajectory estimated by the velocity correction method (method S4: the best method in SL estimation). Finally, SV was calculated as the average of the sensor velocity estimated by method S4. Using the IMU readout, ST, CT, SL, and SV were estimated with 3 ± 3%, 4 ± 3%, 2 ± 6%, and 2 ± 8% relative error, respectively, compared to those obtained from in-lab reference systems' recordings. Other than the bias errors mentioned above, the Bland–Altman plot (Fig. 4) suggested no apparent relationship between the errors of the IMU-based developed methods and the magnitude of the temporal and spatial parameters.

## Discussion

In this study, for the first time, the measurements of the wearable IMUs were used to obtain the temporal and spatial parameters of forward skating (ST, CT, SL, and SV) using various methods, and the results were validated against those obtained by the reference system, i.e., pressure insole and motion-capture cameras. 11 methods were implemented to obtain the temporal events with high accuracy and precision in forward striding in ice skating using wearable IMU readout. Also, four methods were implemented to correct the stride length estimation using IMU readout. The accuracy of temporal and spatial parameters in skating (on average less than 4% of relative error) in this study was comparable with those reported in gait analysis (less than 2% relative error^29,42^). Also, almost the same accuracy in calculating ST has been obtained compared to the other studies on long-track speed skating (3.6%)^11^. Finally, the accuracy of CT and ST obtained by IMU in this study was comparable with those reported in a previous study on ice skating^15^ investigating the accuracy of CT and ST estimated on the real ice (on average, 1% and 2%, respectively). The relatively higher errors in this study compared to the previously mentioned study were due to three factors:

- Lack of a foot-flat period in ice skating: The proposed methods for skater position estimation benefited from velocity correction only once at the end of each measurement trial, while the ZUPT technique in gait analysis can be implemented during the foot-flat period in each gait cycle^6^.
- Lack of familiarity with the synthetic ice: The skaters, even higher calibre skaters, may not have much experience with synthetic ice skating, and thus, they skated less consistently compared to real ice.
- Skating on a shorter skating area requires faster acceleration and deceleration compared to skating on a standard ice rink^31^, where the players tend to skate. Hence, skating on a 14 m-length ice rink could result in more inconsistency in the participants' skating.

Due to the factors above, the performance of the proposed methods implemented for estimating the spatial and temporal parameters of ice skating was more inconsistent compared to gait or real ice skating. Thus, the standard deviation of the obtained errors was larger than those previously reported for gait or real ice skating. Finally, similar to the findings in a gait analysis study^29^, skate acceleration was the most effective time series for on–off ice event detection. Also, to estimate spatial parameters, the suggested methods work more precisely on the pelvis-mounted IMU readouts compared to the readouts from shank- and skate-mounted IMUs. Therefore, only the three IMUs mounted on the skates and pelvis are recommended for estimating the temporal and spatial parameters of ice skaters in forward striding.

### Temporal parameters estimation using skate-mounted IMUs

IMU-based systems have been widely used for temporal event detection in gait analysis and have advantages over other devices such as pressure insoles^29^. The pressure insole has been validated for force measurement and temporal event detection during gait^33,43^, and in this study, we carefully placed them in skates and used them as a reference for short-term trials. Yet, the insoles might not be suitable for long-term skating trials due to (i) their slippage in the skates during long-term dynamic motions and (ii) the inconvenience of carrying a data logger and batteries in a belt connected to the insoles via cables.

The temporal events (SS and BO) detected using IMU readouts showed high accuracy and precision (errors, on average, around one sampling period obtained by T1, T2, and T4 to detect SS and T3 in detecting BO). The methods (T1–T4) that used the upward and forward accelerations of the skate to detect SS and BO were more effective than those that used angular velocities to detect SS and BO (T5, T6, T9, and T10). Implemented methods from gait analysis (T9 and T10) strongly depend on the false positive or false negative detection and the chain of the events detected by these methods. Although these methods were comparably precise in skating event detection, false positive or false negative detection (T9 and T10) of one event influence the proceeding event detections. As a result, the effectiveness of these methods (T9 and T10) requires a repetitive skating pattern leading to repetitive time series all over the trial, which may not be easily achievable, particularly in lower calibre players. In summary, the most effective methods were T1, T2, and T4 in finding SS events and T3, T2, and T1 in detecting BO events in skating when skate-mounted IMUs' readouts were used. Also, the skate-mounted IMUs' readout outperformed the shank-mounted IMUs' readout in temporal events detection (i.e., T1–T4, T7, T8, and T10 compared to the others). As a result, only the two skate-mounted IMUs are recommended for detecting bilateral skating temporal events.

### Spatial parameters estimation using a pelvis-mounted IMU versus skate-mounted IMUs

Four methods were proposed and implemented to remove drifts in the acceleration and velocity time series toward estimating the skater's trajectory using the IMUs recorded on the skater's body. Methods S1–S3 improved the precision of SL estimation by up to 60% compared to no correction scenario. Also, they required only a 0.5-s resting period, making them implementable when there is only one short quiet standing before the motion. On the other hand, Method S4, which removed drifts and corrected the velocity of the pelvis-mounted IMU using the resting periods at the beginning and end of each trial, outperforms the other methods. The relative error obtained by S4 was comparable to those reported in the literature for gait analysis using the ZUPT algorithm^6,29,38^. Furthermore, removing the acceleration's DC offset (calculated at the beginning of the trial in S1) considerably reduced the SL estimation error, and none of the added noise estimation methods, i.e., removing white noise and discreet wavelet transform (DWT) details, in S2 and S3 further reduced the SL estimation errors. This was evident by the Pearson's correlation coefficient of 0.99 between SL obtained by S1 and SL obtained by S2 and S3. In summary, the acceleration obtained by the pelvis-mounted IMU corrected by S4, among the other IMUs and methods, obtained the most precise SL estimation. Also, according to the Bland–Altman plots (Fig. 4), none of the observed estimation errors were a function of the magnitude of the temporal and spatial parameters. In summary, to estimate the temporal and spatial parameters in skating, two skate-mounted IMUs—for temporal event detection—and one pelvis-mounted IMU—for spatial parameter estimation—are recommended.

### Limitations

Development and validation of the proposed methods to estimate the temporal and spatial parameters of ice skating have potential limitations. First, unlike event detection algorithms developed for gait analysis^29^, the norm of acceleration and angular velocity measured by IMUs were less effective in detecting the temporal events of skating. Therefore, acceleration time series were used in the forward or upward directions for temporal event detection, and thus, the obtained accuracy and precision can be affected by the misalignment of the IMUs relative to the skate. Second, extrinsic factors such as temperature, which is different on ice compared to the lab's temperature, can influence the value of IMU readouts. However, they can hardly affect the negative and positive peaks of the IMU readouts^29^) used in the developed event detection methods. Third, our proposed methods were validated only in 14-m trials and forward skating slower than 2.63 m/s. Future studies are needed to investigate the validation of our proposed methods for the measurement of temporal and spatial parameters of ice skating in longer distances, faster skating, and other skating types such as turning. Yet, this study showed that methods S1–S3 by using only 0.5-s resting periods could significantly enhance the stride length estimation precision, at least in short-term (i.e., 8 s) forward striding experiments. Fourth, identification, modelling, and eliminating other sources of error during skating can promise better precision and accuracy in IMU position estimation in miscellaneous skating types in longer experiments. Finally, the effectiveness of IMU pelvis-based methods for temporal event detection can be further investigated in future studies.

## Conclusion

For the first time, novel methods were proposed for estimating the temporal and spatial parameters of ice skating using wearable IMUs and their accuracy and precision were validated against the in-lab reference systems. Among different sensor configurations, the optimal set of IMUs for this purpose consists of two skate-mounted IMUs (for temporal parameters measurement) and one pelvis-mounted IMU (for spatial parameters measurement). The proposed methods estimated the ST, CT, SL, and SV of the ice skaters with relative errors close to those previously reported for gait analysis. The proposed methods detected SS and BO within 0.01-s accuracy using a skate-mounted IMU readout. We improved the SL estimation precision between 53 and 88% using a pelvis-mounted IMU readout. The next step toward developing this wearable technology is to validate the 3D joint angles obtained by the IMUs and validate the measurements on ice. In summary, IMU technology can be utilized for developing metrics for real-time performance assessment of ice hockey players during training sessions and matches. This assessment can potentially help athletes, trainers and coaches by continuously monitoring skating performance and potentially reducing the risk of injuries.

## Data Availability

Generated datasets are available by request to the corresponding author.
